# Chronic Eosinophilic Pneumonia Masquerading as Non-resolving Upper Lobe Pneumonia

**DOI:** 10.7759/cureus.113372

**Published:** 2026-07-25

**Authors:** Jason Liew

**Affiliations:** 1 Respiratory Medicine, Changi General Hospital, Singapore, SGP

**Keywords:** chronic eosinophilic pneumonia, migratory pulmonary infiltrates, non-resolving pneumonia, pragmatic diagnosis, pulmonary eosinophilia, tuberculosis mimics

## Abstract

Chronic eosinophilic pneumonia (CEP) is an uncommon, idiopathic respiratory disorder characterized by the accumulation of eosinophils in the lung parenchyma and alveolar spaces, often accompanied by peripheral blood eosinophilia. Clinically, it frequently presents insidiously with non-specific symptoms such as chronic cough, mimicking infectious pneumonias. In tuberculosis (TB)-endemic regions, upper lobe consolidations routinely anchor clinicians toward mycobacterial workups, resulting in profound diagnostic delays and inappropriate antibiotic use. While bronchoalveolar lavage (BAL) or lung biopsy remains the academic diagnostic gold standard, real-world practice demands clinical flexibility when patients decline invasive interventions. We describe the case of a 65-year-old male non-smoker with a history of diabetes mellitus and hypertension who presented with a one-year history of a waxing and waning chronic cough associated with migratory ("fleeting") pulmonary infiltrates. Due to persistent right upper lobe opacities, he underwent an extensive evaluation at a specialized TB center, which rigorously ruled out active *Mycobacterium tuberculosis* via negative serial sputum smears, molecular assays, and cultures. Subsequent high-resolution chest imaging revealed peripheral-predominant consolidations. Serial laboratory monitoring captured marked systemic peripheral blood eosinophilia (peak absolute eosinophil count: 3.8×10^9/L) and elevated serum immunoglobulin E (729 IU/mL), while systemic vasculitis, fungal infections, and parasitic infestations were systematically excluded. Because the patient strongly declined a recommended diagnostic bronchoscopy with BAL, a pragmatic clinical-radiological diagnosis of CEP was established, and empirical oral corticosteroid therapy (prednisolone 30 mg daily) was initiated. The patient demonstrated an immediate, dramatic clinical resolution within 48 hours, followed by swift normalization of eosinophil kinetics and complete radiological regression of the pulmonary infiltrates within four weeks. This case underscores that a secure, definitive diagnosis of CEP can be safely achieved using longitudinal clinical, radiological, and kinetic laboratory surveillance when gold-standard invasive procedures are declined by the patient and that a rapid, robust therapeutic response to corticosteroids further validates this pragmatic approach once mimicking infectious and autoimmune etiologies are thoroughly excluded.

## Introduction

Eosinophilic lung diseases (ELDs) represent a heterogeneous group of pulmonary disorders characterized by an abnormal accumulation of polymorphonuclear eosinophils within the lung parenchyma, alveolar spaces, and airways [1]. Among these, idiopathic chronic eosinophilic pneumonia (CEP) is a distinct clinical entity marked by an insidious, subacute-to-chronic onset of non-specific respiratory and systemic symptoms, including a persistent cough, dyspnea, and constitutional weight loss [1,2]. Epidemiologically, CEP remains a rare condition, historically accounting for less than 3% of all interstitial lung disease registry entries globally, with a known predilection for women and individuals with a history of atopy or bronchial asthma [2].

Radiologically, CEP classically manifests as bilateral, dense alveolar consolidations with a peripheral-predominant distribution, often described as the "photographic negative" of pulmonary edema [3]. However, these infiltrates can frequently display a waxing, waning, or migratory ("fleeting") behavior over weeks to months, often delaying accurate diagnosis [2,3]. In public health settings with a high clinical suspicion or endemic burden of mycobacterial infections, persistent upper lobe consolidations routinely induce cognitive anchoring, leading clinicians to initial extensive workups to exclude active pulmonary tuberculosis (TB) [3,4]. This clinical overlap routinely results in profound diagnostic delays and the inappropriate use of sequential empirical antimicrobial agents [4].

While the traditional diagnostic gold standard for CEP relies on demonstrating marked alveolar eosinophilia (typically exceeding 25% to 40% on differential cell counts) via bronchoalveolar lavage (BAL) or transbronchial lung biopsy, real-world clinical execution often demands pragmatic flexibility [1,5,6]. A subset of clinical patients are highly risk-averse or have absolute contraindications to invasive flexible bronchoscopy. In these distinct scenarios, a secure and timely diagnosis can be safely established using a constellation of non-invasive parameters, including high-resolution chest imaging demonstrating migratory peripheral opacities, a marked peripheral blood absolute eosinophil count (AEC), elevated serum immunoglobulin E (IgE), and the meticulous exclusion of alternative infectious, fungal, parasitic, or systemic vasculitic mimics [2,5]. Furthermore, CEP is characterized by an exquisite, rapid systemic response to corticosteroid therapy, where clinical and radiological regression within days can serve as retroactive diagnostic verification [5,7].

This case report aims to highlight the diagnostic challenges associated with CEP masquerading as non-resolving upper lobe pneumonia, emphasizing the safety and clinical utility of non-invasive clinical-radiological criteria and longitudinal kinetic laboratory surveillance when invasive gold-standard procedures are declined by the patient.

## Case presentation

History and initial presentation

A 65-year-old male non-smoker with a documented medical history of well-controlled type 2 diabetes mellitus (baseline hemoglobin A1c: 6.8%), essential hypertension, and hyperlipidemia presented to our tertiary respiratory service in June 2026. He sought a secondary evaluation for a persistent chronic cough and highly anomalous, recurrent thoracic imaging abnormalities.

The patient's clinical symptoms originally manifested in August 2025, with a one-month history of an intermittent cough productive of clear sputum, accompanied by an unintentional 2 kg weight loss. He explicitly denied hemoptysis, fever, nocturnal diaphoresis, orthopnea, or gastroesophageal reflux symptoms. He was initially treated empirically by a primary care physician with two separate courses of oral antibiotics, which yielded no clinical resolution.

Owing to unremitting symptoms, he presented to an emergency department where an initial chest radiograph demonstrated patchy, multi-lobar consolidations spanning the left pericardiac and bilateral lower lung zones, associated with a trace left pleural effusion. He was managed under a presumptive diagnosis of community-acquired pneumonia. A surveillance chest radiograph performed in October 2025 demonstrated complete spontaneous resolution of the lesions, aligning with a temporary abatement of his cough.

Diagnostic delay and mycobacterial workup

The cough recurred in late October 2025 and persisted continuously through January 2026. In March 2026, a repeat chest radiograph revealed a new, dense, mass-like consolidation isolated entirely to the right upper lobe. Due to the chronic nature of his structural lesions and the anatomical predilection for the upper lobe, the patient was urgently referred to a specialized TB control center to rule out active TB.

At the specialized center, the patient underwent a rigorous microbiological and immunological investigation. A Mantoux tuberculin skin test was completely non-reactive (0 mm induration). Serial induced sputum evaluations, including two separate acid-fast bacilli smears, automated mycobacterial nucleic acid amplification testing (GeneXpert PCR), and standard mycobacterial cultures, consistently demonstrated no evidence of *Mycobacterium tuberculosis*.

Given the non-resolving upper lobe consolidation, the center recommended a navigational bronchoscopy with transbronchial lung biopsy and BAL. However, the patient, being highly risk-averse, declined invasive airway intervention and self-referred to our clinic.

Investigations and longitudinal laboratory tracking

Upon presentation to our institution, longitudinal laboratory surveillance was conducted by compiling historical records and current serum profiles. This revealed an anomalous, progressive shift in the patient's white blood cell differential. The peripheral blood AEC demonstrated severe systemic progression, peaking concurrently with his right upper lobe structural consolidation. Total serum IgE levels were also markedly elevated.

To safely initiate systemic immunosuppressive therapy, an extensive exclusionary panel was executed to rule out systemic vasculitis, fungal infections, and parasitic infestations. The full timeline of the patient's longitudinal hematological kinetics, immunology, and comprehensive diagnostic exclusionary workup is compiled in Table 1.

**Table 1:** Longitudinal hematological kinetics and diagnostic exclusionary workup.

Test Name	Reference Range	August 2025 (Baseline)	March 2026 (Peak)	Apr-26	June 2026 (Pre-treatment)	July 2026 (Post-treatment)
Hematology & Kinetics						
Hemoglobin (g/dl)	13.0- 17.0	13.7	16	15	14.6	15
WBC Count (X10^3/uL)	4.0-10.0	10.5	13.6	12	11.5	8
Platelet Count (X10^3/uL)	150-400	388	344	244	235	256
Absolute Eosinophil Count (x10^9/L)	0.04 - 0.40	0.5	3.8	3.58	2.9	0.05
Immunology & Autoimmune						
Total Serum IgE (IU/mL)	< 100	—	—	691	729	—
Hemoglobin A1c (%)	4.0 - 6.0	—	—	—	6.8	—
c-ANCA (PR3)	Negative	—	—	—	Negative	—
p-ANCA (MPO)	Negative	—	—	—	Negative	—
Antinuclear Antibodies (ANA)	Negative	—	—	—	Negative	—
Microbiology & Surveillance						
Sputum AFB Smears (x2)	Negative	—	Negative	—	—	—
Sputum GeneXpert M. tuberculosis PCR	Negative	—	Negative	—	—	—
Sputum Mycobacterial Culture	Negative	—	Negative	—	—	—
Stool Ova Cysts & Parasites (x3)	Negative	—	—	—	Negative	—
HIV Screen	Non-reactive	—	—	—	Negative	—
Hepatitis B Surface Antigen	Non-reactive	—	—	—	Negative	—
Hepatitis C Antibody	Non-reactive	—	—	—	Negative	—

Radiological surveillance

**Figure 1:**
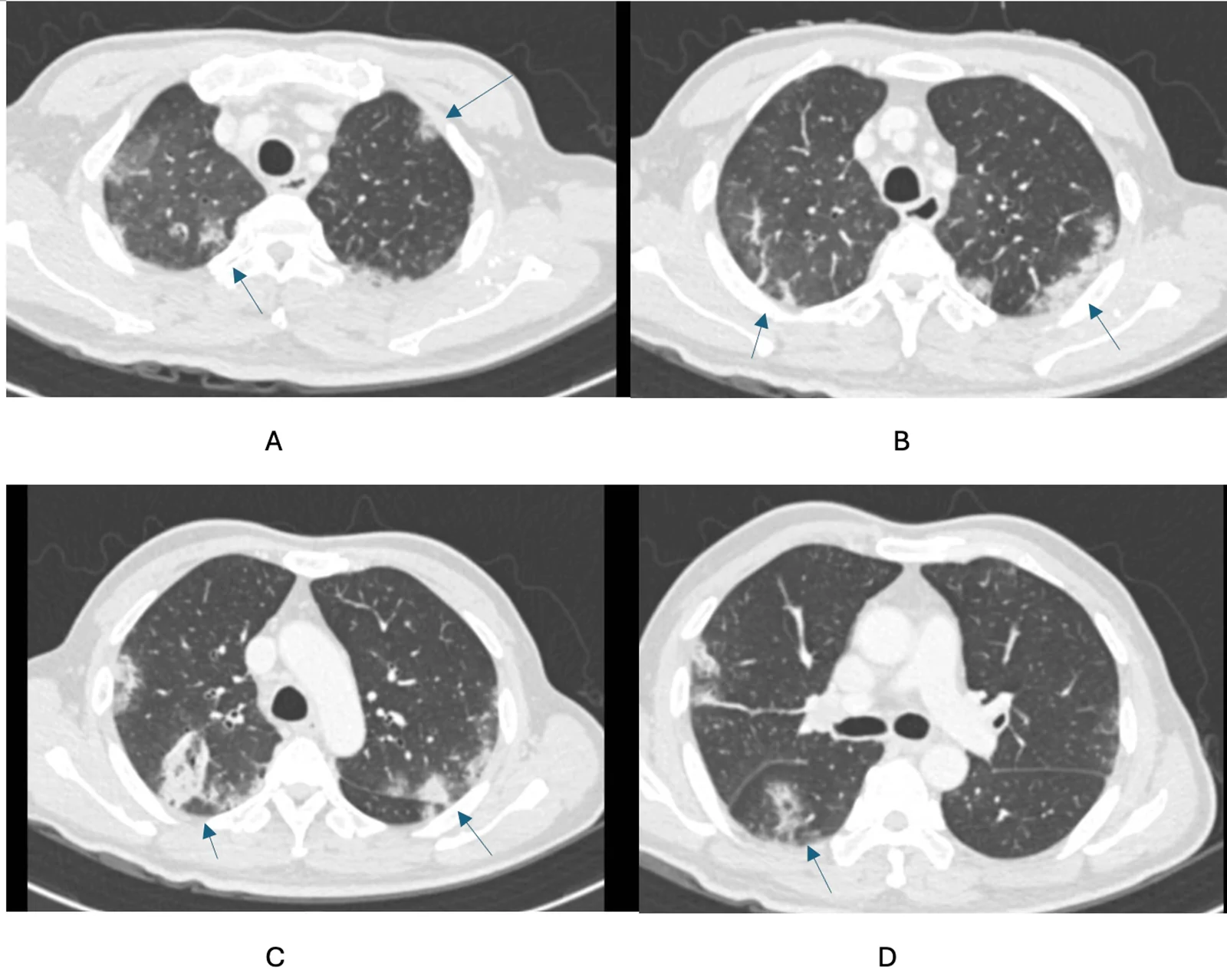
Axial high-resolution computed tomography (HRCT) of the thorax demonstrating widespread, peripheral-predominant consolidations characteristic of chronic eosinophilic pneumonia. Panel A (Axial view at the lung apex) Panel B (Axial view in the superior upper region) Panel C (Axial view at the level of the aortic arch) Panel D (Axial view at the level of the carina)

A high-resolution computed tomography scan of the thorax confirmed dense, multi-lobar upper lobe consolidations displaying a peripheral-predominant distribution and focal nodular configurations, alongside mild mediastinal lymphadenopathy (Figure 1).

**Figure 2:**
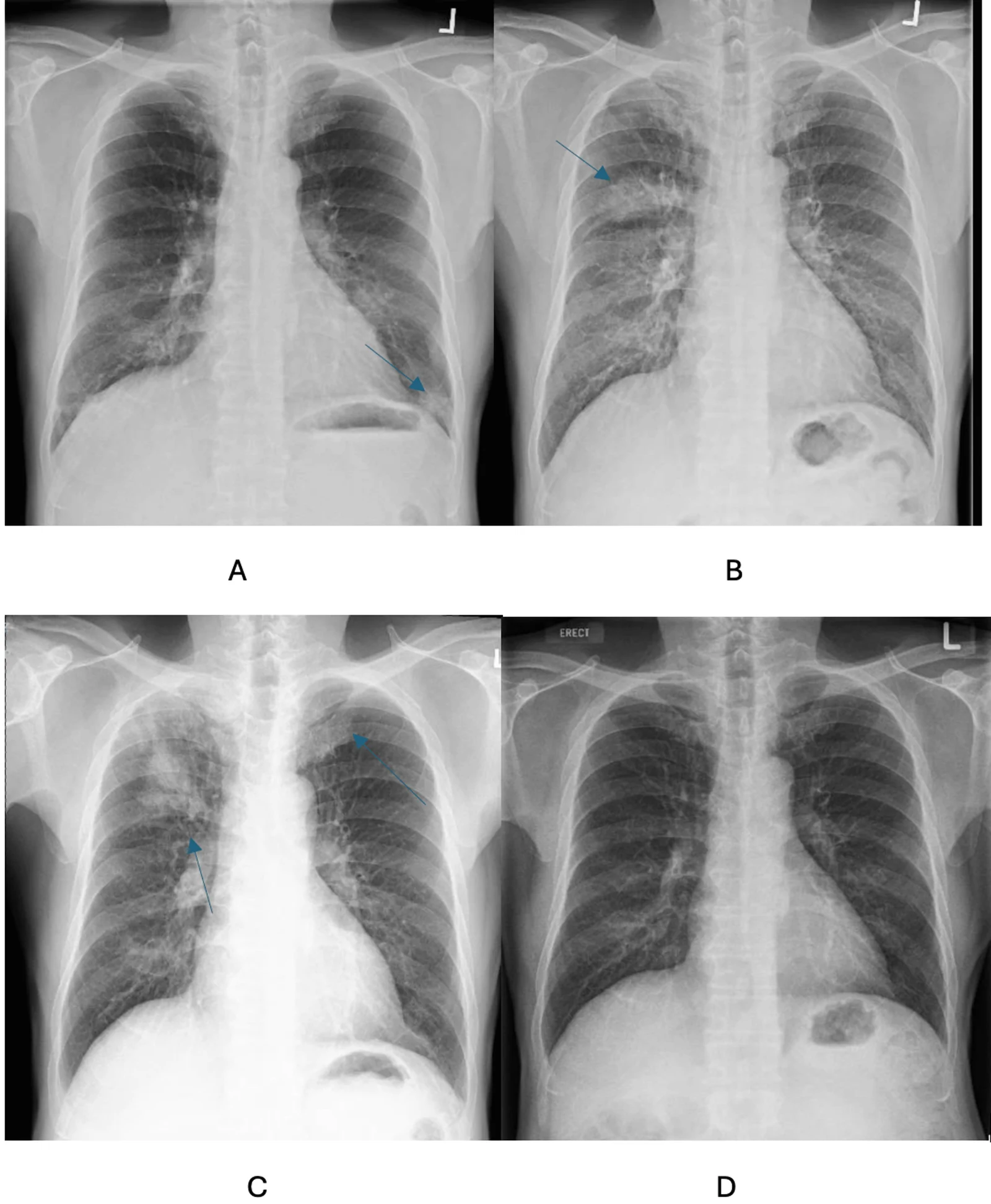
Serial chest radiographs demonstrating the classic migratory ("fleeting") pattern and rapid therapeutic responsiveness of chronic eosinophilic pneumonia (CEP). Panel A (August 2025): Initial baseline chest radiograph obtained at early symptom onset, demonstrating patchy, ill-defined alveolar consolidations predominantly localized to the left pericardiac and bilateral lower lung zones. Panel B (March 2026): Follow-up chest radiograph taken following a recurrence of symptoms, capturing a striking anatomical migration of the consolidations. The previous lower-zone infiltrates have spontaneously cleared, replaced by a dense, mass-like consolidation isolated to the right upper and middle lung fields. This persistent upper lobe presentation mimicked localized infectious pathologies and triggered an urgent clinical referral to the National Tuberculosis Control Center (NTBCC). Panel C (June 2026): Pre-treatment chest radiograph demonstrating further migratory kinetics, with the development of new, bilateral upper lobe reticulonodular opacities, apical pleural thickening, and fleeting infiltrates. Panel D (July 2026): Surveillance chest radiograph obtained just three weeks after the initiation of empirical systemic corticosteroid therapy (oral prednisolone 30 mg daily). The image demonstrates complete, near-total radiological resolution of all bilateral upper lobe consolidations, confirming the classic, exquisite steroid responsiveness characteristic of idiopathic CEP.

Sequential chest radiographs tracking the structural progression captured a classic migratory pattern (Figure 2). A pre-treatment chest radiograph in June 2026 demonstrated bilateral upper lobe opacities, more significant on the right side, confirming a waxing, waning, and distinctly "fleeting" clinical-radiological phenotype. The final chest radiograph after the initiation of steroids shows immediate resolution of opacity. 

Management and therapeutic trial

Following comprehensive multidisciplinary counseling, the vital diagnostic utility of a bronchoscopy with BAL was re-emphasized to definitively quantify alveolar eosinophilia. The patient maintained a strict, risk-averse preference against invasive interventions. Balancing the strong clinical-radiological evidence against the patient’s preferences, a pragmatic decision was made to initiate an empirical therapeutic trial of oral corticosteroids for suspected idiopathic CEP.

The primary clinical risk discussed was the potential for systemic corticosteroids to unmask or accelerate an occult infection, particularly TB or strongyloidiasis; this was mitigated by the exhaustive baseline exclusionary screen detailed in Table 1. Metabolic co-prescription was established using oral omeprazole (20 mg daily) for gastric cytoprotection, alongside a structured regimen of home capillary blood glucose monitoring to protect against steroid-induced glycemic crises. The patient was started on oral prednisolone at an empirical dose of 30 mg daily (~0.5 mg/kg/day) for an initial duration of four weeks.

Outcome and follow-up

The patient demonstrated an exquisite, rapid response to the therapeutic trial. At his three-week clinical review, he reported complete resolution of his chronic cough and dyspnea, accompanied by a total return to his baseline functional energy levels.

Objective monitoring parameters verified a complete systemic response. His peripheral blood absolute eosinophil count dropped dramatically from a pre-treatment value of 2.90×109/L to a fully suppressed value of 0.05×109/L (Table 1). Follow-up chest radiography demonstrated near-total radiological regression, with the bilateral apical opacities fully resolving. Home capillary glucose monitoring confirmed excellent metabolic stability without steroid-induced glycemic spikes, preserving his stable diabetic profile.

Given the successful therapeutic mirroring, the clinical diagnosis of CEP was retroactively confirmed. To prevent the high recurrence rates characteristic of CEP, a strict, prolonged steroid taper was instituted: prednisolone 30 mg daily for weeks 1-4 (completed), followed by 20 mg daily for weeks 5-8, and 10 mg daily for weeks 9-12. The patient remains under close surveillance with scheduled clinical reviews and serial chest radiographs at each taper transition.

## Discussion

The clinical journey of this patient highlights several critical diagnostic and therapeutic paradigms in the contemporary management of idiopathic CEP. Chief among these is the clinical utility of pragmatic diagnostic criteria when gold-standard procedures cannot be executed. While traditional academic guidelines dictate that a definitive diagnosis of CEP relies on demonstrating marked alveolar eosinophilia, typically exceeding 25% to 40% on differential cell counts via BAL or transbronchial lung biopsy, real-world clinical practice frequently demands diagnostic flexibility [5,8]. When a patient is highly risk-averse or presents with absolute contraindications to invasive flexible bronchoscopy, a secure and timely diagnosis can be safely established using a constellation of non-invasive parameters. As demonstrated in this case, these parameters include a chronic duration of respiratory symptoms, a markedly elevated peripheral blood absolute eosinophil count, elevated serum IgE, and high-resolution chest imaging demonstrating peripheral-predominant, migratory consolidations [2,5].

When benchmarked against the broader body of documented CEP literature, this case shares classic hallmarks while exhibiting important atypical deviations that hold diagnostic significance. In alignment with traditional cohorts described since Carrington et al., our patient presented with subacute respiratory distress, constitutional systemic symptoms, marked peripheral blood eosinophilia [8] and a rapid, dramatic response to systemic corticosteroids [2,5]. However, key radiologic and morphological distinctions were observed. Classic CEP typically manifests as non-segmental, peripheral-predominant ground-glass opacities or dense consolidations-often termed the "photographic negative" of pulmonary edema [2,5]. In contrast, our patient’s imaging revealed localized internal necrosis/small cavitation within the area of consolidation. While cavitation is exceedingly rare in classic CEP, its presence here can be logically attributed to severe, localized eosinophil-mediated tissue destruction resulting from sustained degranulation of cytotoxic proteins (e.g., major basic protein and eosinophil peroxidase) during prolonged, uninhibited inflammation prior to steroid initiation. Furthermore, while conditions like allergic bronchopulmonary aspergillosis (ABPA) frequently present with migratory opacities, the distinct absence of central bronchiectasis and negative fungal markers helped differentiate our case from ABPA and invasive pulmonary aspergillosis, reinforcing idiopathic CEP as the unifying diagnosis.

A significant challenge in the early recognition of CEP is the phenomenon of cognitive anchoring, particularly in geographic regions with a high endemic burden or significant public health focus on mycobacterial infections. Persistent upper lobe consolidations routinely bias clinicians toward infectious etiologies, frequently leading to extensive workups to exclude active pulmonary TB [3,4]. This clinical overlap routinely results in profound diagnostic delays, sequential courses of inappropriate empirical antimicrobial agents, and prolonged patient anxiety [4]. To avoid this diagnostic trap, the identification of a "fleeting" or migratory pattern on serial chest radiographs must serve as an immediate clinical pivot. Recognizing that consolidations are melting away in one lung zone while simultaneously emerging in another should prompt clinicians to expand their differential diagnosis beyond classic infectious pneumonias to encompass non-infectious, eosinophilic, or organizing lung pathologies [2,3].

However, the initiation of empirical, high-dose systemic corticosteroid therapy for suspected ELDs must always be preceded by a rigorous baseline exclusionary screening [9,10]. Because corticosteroids possess potent immunosuppressive properties, their empirical administration carries the catastrophic potential to mask an occult pulmonary malignancy or induce a life-threatening hyperinfection if an underlying infectious process is missed. Therefore, before exposing a patient to systemic steroids, clinicians must perform a diligent screening panel. This baseline workup must include multi-sample mycobacterial cultures, highly sensitive molecular polymerase chain reaction assays, autoimmune serologies to rule out systemic vasculitis, and comprehensive parasitic or stool screens to rule out strongyloidiasis and other helminthic hyperinfection mimics.

Once an alternative infectious or autoimmune mimic is systematically excluded, the patient's response to systemic corticosteroids can provide profound diagnostic utility through therapeutic response mirroring. Idiopathic CEP is characterized by an exquisite and rapid sensitivity to corticosteroid therapy, where a dramatic clinical and radiological resolution within 48 to 72 hours acts as retroactive diagnostic verification [5-7]. Nevertheless, achieving an immediate initial response does not signal the completion of therapy. Due to the disease's high baseline propensity for relapse upon premature steroid withdrawal, a prolonged, highly structured, and gradual taper spanning several months is mandatory to ensure sustained clinical remission and complete radiological regression [1].

## Conclusions

In conclusion, this case demonstrates that CEP can be safely diagnosed without invasive BAL by relying on migratory consolidations, severe peripheral eosinophilia, and elevated serum IgE. Clinicians must recognize "fleeting" opacities on serial imaging as a critical diagnostic pivot to break cognitive anchoring toward TB in endemic regions. Furthermore, while rapid clinical resolution under corticosteroids provides retroactive diagnostic confirmation, rigorous baseline exclusionary screening for infectious and autoimmune mimics is mandatory before initiating therapy, followed by a prolonged, structured steroid taper to prevent relapse.
